# The Aedes aegypti Domino Ortholog p400 Regulates Antiviral Exogenous Small Interfering RNA Pathway Activity and *ago-2* Expression

**DOI:** 10.1128/mSphere.00081-20

**Published:** 2020-04-08

**Authors:** Melanie McFarlane, Floriane Almire, Joy Kean, Claire L. Donald, Alma McDonald, Bryan Wee, Mathilde Lauréti, Margus Varjak, Sandra Terry, Marie Vazeille, Rommel J. Gestuveo, Isabelle Dietrich, Colin Loney, Anna-Bella Failloux, Esther Schnettler, Emilie Pondeville, Alain Kohl

**Affiliations:** a MRC-University of Glasgow Centre for Virus Research, Glasgow, Scotland; b Arboviruses and Insect Vectors Unit, Department of Virology, Institut Pasteur, Paris, France; c Usher Institute for Population Health Sciences & Informatics, University of Edinburgh, Edinburgh, United Kingdom; d Division of Biological Sciences, University of the Philippines Visayas, Miagao, Philippines; Icahn School of Medicine at Mount Sinai

**Keywords:** RNA interference, *ago-2*, arbovirus, innate immunity, mosquito, p400

## Abstract

Arboviruses are pathogens of humans and animals. A better understanding of the interactions between these pathogens and the arthropod vectors, such as mosquitoes, that transmit them is necessary to develop novel control measures. A major antiviral pathway in the mosquito vector is the exogenous small interfering RNA (exo-siRNA) pathway, which is induced by arbovirus-derived double-stranded RNA in infected cells. Although recent work has shown the key role played by Argonaute-2 (Ago-2) and Dicer-2 (Dcr-2) in this pathway, the regulatory mechanisms that govern these pathways have not been studied in mosquitoes. Here, we show that the Domino ortholog p400 has antiviral activity against the alphavirus Semliki Forest virus (*Togaviridae*) both in Aedes aegypti-derived cells and *in vivo*. Antiviral activity of p400 was also demonstrated against chikungunya virus (*Togaviridae*) and Bunyamwera virus (*Peribunyavirida*e) but not Zika virus (*Flaviviridae*). p400 was found to be expressed across mosquito tissues and regulated *ago-2* but not *dcr-2* transcript levels in A. aegypti mosquitoes. These findings provide novel insights into the regulation of an important aedine exo-siRNA pathway effector protein, Ago-2, by the Domino ortholog p400. They add functional insights to previous observations of this protein’s antiviral and RNA interference regulatory activities in Drosophila melanogaster.

**IMPORTANCE** Female Aedes aegypti mosquitoes are vectors of human-infecting arthropod-borne viruses (arboviruses). In recent decades, the incidence of arthropod-borne viral infections has grown dramatically. Vector competence is influenced by many factors, including the mosquito’s antiviral defenses. The exogenous small interfering RNA (siRNA) pathway is a major antiviral response restricting arboviruses in mosquitoes. While the roles of the effectors of this pathway, Argonaute-2 and Dicer-2 are well characterized, nothing is known about its regulation in mosquitoes. In this study, we demonstrate that A. aegypti p400, whose ortholog Domino in Drosophila melanogaster is a chromatin-remodeling ATPase member of the Tip60 complex, regulates siRNA pathway activity and controls *ago-2* expression levels. In addition, we found p400 to have antiviral activity against different arboviruses. Therefore, our study provides new insights into the regulation of the antiviral response in A. aegypti mosquitoes.

## Abstract (precis)

Female Aedes aegypti mosquitoes are vectors of human-infecting arthropod-borne viruses (arboviruses). In recent decades, the incidence of arthropod-borne viral infections has grown dramatically. Vector competence is influenced by many factors, including the mosquito’s antiviral defenses. The exogenous small interfering RNA (siRNA) pathway is a major antiviral response restricting arboviruses in mosquitoes. While the roles of the effectors of this pathway, Argonaute-2 and Dicer-2 are well characterized, nothing is known about its regulation in mosquitoes. In this study, we demonstrate that A. aegypti p400, whose ortholog Domino in Drosophila melanogaster is a chromatin-remodeling ATPase member of the Tip60 complex, regulates siRNA pathway activity and controls *ago-2* expression levels. In addition, we found p400 to have antiviral activity against different arboviruses. Therefore, our study provides new insights into the regulation of the antiviral response in A. aegypti mosquitoes.

## INTRODUCTION

Arthropod-borne viruses (arboviruses) are transmitted to susceptible mammalian hosts through the bite of infected arthropod vectors, such as mosquitoes. This group of viruses includes those of medical and veterinary importance, such as chikungunya virus (CHIKV) (Alphavirus; *Togaviridae*), dengue virus (DENV) (Flavivirus; *Flaviviridae*), Zika virus (ZIKV) (*Flavivirus*; *Flaviviridae*), and Rift Valley fever virus (RVFV) (recently reclassified into Phlebovirus; *Phenuiviridae*) (1–6). Given the medical and economic impacts of arboviruses, viral interactions with mosquitoes and the impact on transmission remain important areas of research. New strategies to interfere with arbovirus transmission involve genetically modified mosquitoes, including making mosquitoes more resistant to arboviruses, as well as Wolbachia-endosymbiont-based approaches (7–15). Arboviruses infect and replicate in both mammalian host and vector host cells; as a consequence, they are detected by both mammalian and arthropod immune systems. Compared to the wealth of knowledge on the mammalian antiviral immune response, the arthropod response is not well characterized, yet it could provide important targets for novel vector control measures. Research on this topic has established that the key player in the antiviral immune response in mosquito vectors is the exogenous small interfering RNA (exo-siRNA) pathway (8, 16–19). This pathway is activated by the recognition of long viral double-stranded RNA (dsRNA) by the endo-RNase Dicer-2 (Dcr-2). The effector protein Dcr-2 cleaves viral dsRNA into 21-nucleotide (nt) virus-derived siRNAs (vsiRNAs), which are then loaded into the RNA-induced silencing complex (RISC). One key protein within RISC, Argonaute-2 (Ago-2), binds the siRNA and is believed to unwind the siRNA duplex and use one of the strands as a guide to specifically recognize complementary RNAs, targeting these for degradation. In the case of viral RNAs (such as genomes, antigenomes, or mRNAs), this results in an inhibition of virus replication. The antiviral activity mediated by the exo-siRNA pathway of vector mosquitoes has been demonstrated through the silencing or elimination/absence of effector proteins (such as Ago-2 or Dcr-2), which results in an upregulation of arbovirus replication. It has been shown to be active against arboviruses of all major families or orders, alphaviruses (20–29), flaviviruses (30–34), and bunyaviruses (35–37).

Although the importance of the key exo-siRNA pathway components, such as Ago-2, has been established, the regulation of this antiviral response, such as effector protein expression, activity, and activation, remains poorly understood. Given its attractiveness as a target for immunity-based control strategies in vectors, it is important that all of the components, including regulatory mediators, of the exo-siRNA pathway are better understood. In this study, we sought to identify new components of the antiviral exo-siRNA pathway with the aim of understanding how the pathway functions and how it is regulated. We show that the Aedes aegypti Domino ortholog p400 is expressed at different levels across mosquito tissues and is an antiviral factor, since silencing of the gene enhanced replication of the mosquito-borne alphavirus Semliki Forest virus (SFV). We confirmed that p400 not only acts on SFV but also on the SFV-related alphavirus CHIKV, as well as Bunyamwera virus (BUNV), a member of the *Peribunyaviridae* family. However, p400 did not show antiviral activity against ZIKV. In addition, we have identified p400 as a regulator of the exo-siRNA pathway activity, possibly by controlling *ago-2* but not *dcr-2* transcript levels *in vivo*. Thus, our results hint that this protein may exert antiviral activity through regulating the exo-siRNA pathway, although other antiviral pathways potentially regulated by p400 cannot be excluded. These findings help to further understand the regulation of the antiviral exo-siRNA pathway.

## RESULTS

### p400 is expressed in female mosquitoes.

**FIG 1:**
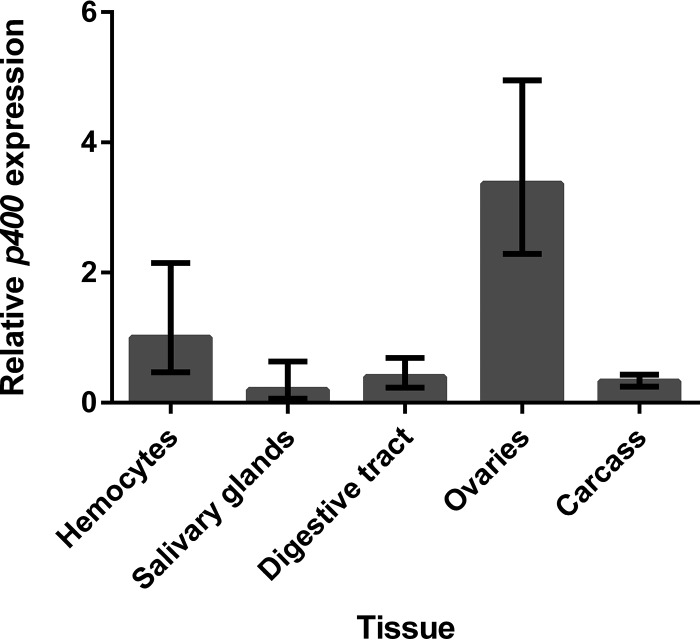
Detection of *p400* transcripts in tissues of NBF A. aegypti females. Presence of *p400* transcripts was determined in hemocytes, salivary glands, digestive tracts, ovaries, and carcasses of NBF females by RT-qPCR using the 2^−ΔΔ^*^CT^* method and expression in hemocytes as the reference sample. Bars represent the fold change in gene expression from 3 independent biological replicates (pools of 25 digestive tracts or ovaries, pools of 60 salivary glands, and pools of perfused hemocytes from 70 females per replicate). Error bars show the minimum and maximum fold change.

Previous studies have reported the role of Domino in the antiviral response in Drosophila melanogaster (38). We therefore wanted to determine if the A. aegypti Domino ortholog, p400, has a similar role in mosquitoes. To determine if p400 was expressed in tissues relevant to arbovirus-mosquito interactions, we first investigated the presence of *p400* transcripts across mosquito tissues. For this, individual tissues were dissected and hemocytes perfused from non-blood-fed (NBF) female mosquitoes. RNA was extracted and transcript levels analyzed by reverse transcription-quantitative PCR (RT-qPCR). As shown in Fig. 1, *p400* transcripts were detected in hemocytes and all tissues tested. The expression levels of *p400* varied across tissues, with low levels in salivary glands and high levels in ovaries.

**FIG 2:**
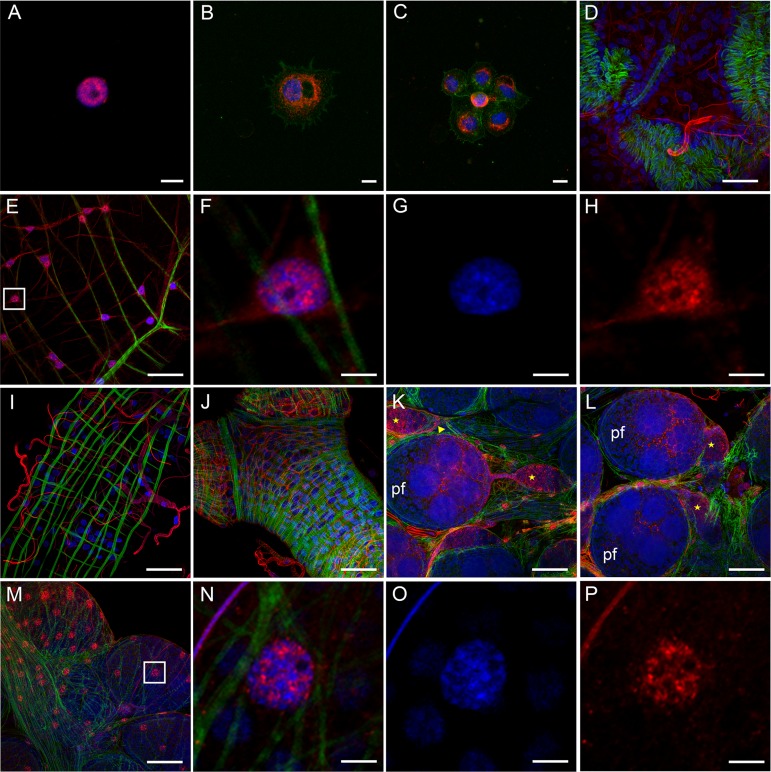
Detection of p400 protein in tissues of NBF A. aegypti females. Expression of p400 was analyzed in perfused hemocytes (A to C), salivary glands (D), digestive tracts (E to I), and ovaries (J to P) by immunofluorescence assay using an anti-p400 antibody. The signal was determined using an Alexa Fluor 568 goat anti-mouse IgG (H+L) (red). Nuclei are stained by DAPI (blue signal), and F-actin is stained by phalloidin 488 (green signal). Images were acquired on a Zeiss LSM 710 inverted confocal microscope with 40×, 63×, and 100× oil immersion objectives. Scale bars are 5 μm (A to C, F to H, and N to P) and 40 μm (D, E, and I to M). (A) Perfused prohemocyte. (B and C) Perfused differentiated hemocytes. (D) Salivary glands. (E) Crop. (F to H) Enlargement of panel E. (I) Midgut. (J) Oviduct. (K and L) Ovarioles showing the germarium and primary follicle (pf). (M) Ovarioles surrounded by the ovarian sheath. (N to P) Enlargement of panel M. Yellow star, developing cyst in the germarium; yellow arrowhead, germ line stem cells. The cystoblast is located between the germ line stem cells and the developing cyst. Images shown are representative of a minimum of 10 tissues per experiment from 3 independent experiments.

The presence of p400 was also investigated by immunofluorescence. Consistent with our RT-qPCR results, p400 was detected in hemocytes and several sampled tissues (Fig. 2). p400 was expressed in all perfused hemocytes. It was restricted to the nucleus in prohemocytes (Fig. 2A), which are small and spherical cells, with a high nuclear/cytoplasmic ratio (39). It was expressed in the nucleus as well as in the cytoplasm of differentiated hemocytes or granulocytes (Fig. 2B and C), which are bigger cells with filopodia and a low nuclear/cytoplasmic ratio (39). In tissues, p400 was detected in the nucleus of cells in the digestive tract, such as in the crop (Fig. 2E to H), in the ovarian sheath surrounding the whole ovary and oviduct (Fig. 2J and M to P), and in the ovariolar sheath surrounding each ovariole (Fig. 2K and L). In ovarioles, p400 was expressed in some of the somatic cells forming the epithelium surrounding the germarium and the primary previtellogenic follicle (Fig. 2K and L). In the germarium, p400 was also strongly expressed in the nucleus of differentiating germ cells of the developing cyst (which will give rise to the secondary follicle) as well as in the germ line stem cells and to a lesser extent in the cystoblast germ cells (Fig. 2K). We also found p400 in the tracheal cells surrounding various tissues (Fig. 2D and I to K).

### p400 is antiviral against SFV in A. aegypti females.

**FIG 3:**
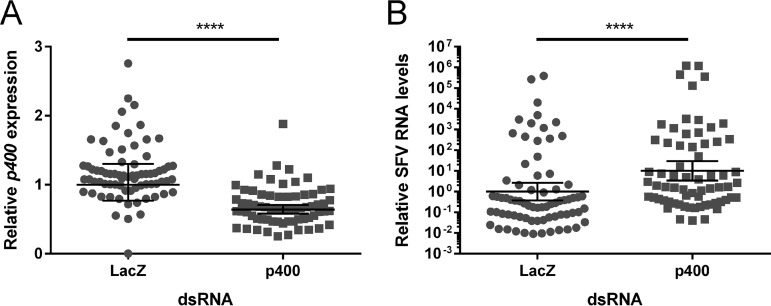
*p400* knockdown significantly enhances replication of SFV in A. aegypti females. *p400* and SFV expression in whole dsLacZ- or dsp400-injected females 3 days after an SFV4-infected blood meal were analyzed by RT-qPCR. Normalized expression-per-sample values were calculated as described by Taylor et al. (78) in order to obtain normalized expression values, relative to the ribosomal S7 transcript as reference, with a geomean of 1 for the dsLacZ control group. Bars represent the geomean and 95% confidence intervals from *n* = 73 dsLacZ and *n* = 66 dsp400. The results were analyzed using a Mann-Whitney test using log_2_-normalized expression values. This experiment is representative of two independent biological replicates (second replicate shown in Fig. S1). (A) *p400* transcript levels are significantly reduced in dsp400-injected females compared to that in dsLacZ-injected females (mean fold change, 1.7; geomean fold change, 1.6; ****, *P* < 0.0001). (B) SFV4 titers are significantly higher in dsp400-injected females than in dsLacZ-injected ones (mean fold change, 5.3; geomean fold change, 10.1; ****, *P* < 0.0001). A base 10 log scale is used for the *y* axis.

Yasunaga et al. previously reported that Domino has antiviral activity against West Nile virus (WNV) and vesicular stomatitis virus (VSV) in Drosophila DL1 cells (38). Therefore, we sought to determine if the Domino ortholog p400 has similar antiviral activity in A. aegypti females. For this, we assessed whether p400 could be silenced *in vivo* and determined the effect of this knockdown on viral infection using the model alphavirus SFV. Female A. aegypti mosquitoes were injected intrathoracically with *p400*-targeting dsRNA (dsp400) or control *lacZ*-targeting dsRNA (dsLacZ). Mosquitoes were fed with a blood meal containing SFV4 at 4 days post-dsRNA injection (pdi). At 3 days postinfection (pi), RNA was extracted from whole females. Knockdown efficiency and virus genome levels were determined by RT-qPCR. The knockdown of *p400* transcript expression was confirmed (Fig. 3A) and resulted in a significant increase in SFV RNA levels in whole females (Fig. 3B), confirming the role of p400 in inhibiting SFV replication in A. aegypti female mosquitoes. The results were repeated in a second independent experiment (see Fig. S1 in the supplemental material).

10.1128/mSphere.00081-20.1 FIG S1 *p400* knockdown effect on SFV replication and *ago-2* expression in A. aegypti females. *p400*, SFV, and *ago-2* in whole dsLacZ- or dsp400-injected females 3 days after an SFV4-infected blood meal were analyzed by RT-qPCR. Normalized expression-per-sample values were obtained as described by Taylor et al. (78) in order to obtain normalized expression values, relative to the ribosomal S7 transcript as a reference, with a geomean of 1 for the dsLacZ control group. Bars represent the geomean and 95% confidence interval from *n* = 39 females for dsLacZ and *n* = 43 for dsp400. The results were analyzed using a Mann-Whitney test using log_2_-normalized expression values. The figure represents results from the second independent biological replicate (first replicate shown in Fig. 3 and 6). (A) *p400* knockdown efficiency. *p400* transcript levels are significantly reduced in dsp400-injected females compared to those in dsLacZ-injected females (mean fold change, 2.1; geomean fold change, 1.9; ****, *P* < 0.0001). (B) Effect of *p400* knockdown on SFV infection in whole female*s*. SFV4 titers are significantly higher in dsp400-injected females than in dsLacZ-injected ones (mean fold change, 138.9; geomean fold change, 2.7; *P* = 0.0188; *, *P* < 0.05). (C) Effect of *p400* knockdown on *ago-*2 transcript levels. *ago-2* transcript levels are significantly reduced in dsp400-injected females compared to those in dsLacZ-injected ones (mean fold change, 1.3; geomean fold change, 2.5; *P* = 0.0109; *, *P* < 0.05). Download FIG S1, TIF file, 0.1 MB. Copyright © 2020 McFarlane et al. 2020 McFarlane et al. This content is distributed under the terms of the Creative Commons Attribution 4.0 International license.

### p400 shows antiviral activity against SFV, CHIKV, and BUNV.

**FIG 4:**
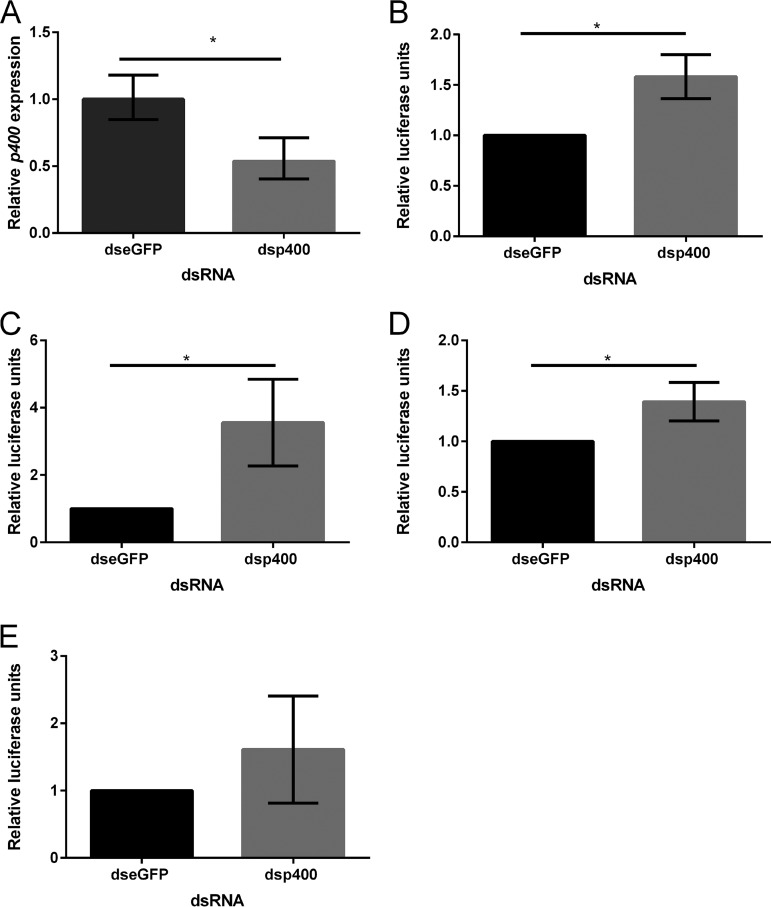
*p400* knockdown significantly enhances replication of SFV, CHIKV, and BUNV but not ZIKV. (A) *p400* knockdown efficiency in Aag2 cells was analyzed by RT-qPCR using the 2^−ΔΔ^*^CT^* method and *p400* expression calculated relative to eGFP dsRNA transfection. Bars show the fold change in gene expression from 3 independent experiments (3 wells in each independent experiment, with an average of three wells per condition/experiment used for statistical analysis). Error bars represent the minimum and maximum fold change. The results were analyzed by a one-sample *t* test. *p400* transcript levels are significantly reduced after dsp400 transfection (*P* = 0.039; *, *P* < 0.05). The effect of p400 knockdown on SFV-*FFLuc* (B), CHIKV-2SG-*FFLuc* (C), BUNV-NLuc (D), and ZIKV-NLuc (E) was assessed by a luciferase assay. Bars show the means of the results from 3 independent experiments (3 wells in each independent experiment, with the average of three replicates per condition/experiment used for statistical analysis). Values were calculated relative to the control eGFP dsRNA-transfected sample, which was set to 1. Statistical significance was determined by performing a one-sample *t* test (SFV *P* = 0.0129, CHIKV *P* = 0.0286, and BUNV *P* = 0.0257; *, *P* < 0.05). Error bars show the standard error of mean.

To determine if the antiviral action of p400 was specific to SFV or if it has broader antiviral activity, we tested the effect of p400 knockdown on three other arboviruses belonging to different families, the SFV-related alphavirus CHIKV, the bunyavirus BUNV, and the flavivirus ZIKV. For this experiment, A. aegypti-derived Aag2 cells were used, and the effect of the p400 knockdown on SFV was used as a positive control. The reporter viruses used for these experiments were CHIKV-2SG-*FFLuc* (*FFLuc*, firefly luciferase expressing), BUNV-NLuc and ZIKV-NLuc (both NLuc, Nano luciferase expressing), and SFV4(3H)-*FFLuc* (also firefly luciferase expressing) (25, 29, 35, 40). Aag2 cells were transfected with dsp400-targeting or control dsRNA-targeting enhanced green fluorescent protein (dseGFP). Cells were infected with CHIKV-2SG-*FFLuc*, BUNV-NLuc, ZIKV-NLuc, or SFV4(3H)-*FFLuc* at 24 h posttransfection (pt). SFV- and CHIKV-infected cells were lysed 24 hpi, BUNV-infected cells were lysed at 48 hpi, and ZIKV-infected cells were lysed at 72 hpi, and luciferase activity was determined. The time posttransfection at which cells were collected was chosen based on previous studies (29, 34, 35, 41). Knockdown of *p400* (Fig. 4A) resulted in a significant increase in SFV (Fig. 4B), confirming our results obtained *in vivo*, as well as on CHIKV (Fig. 4C) and BUNV (Fig. 4D) replication. However, p400 knockdown had no significant effect on ZIKV, possibly due to higher variation of luciferase expression across replicates for this virus (Fig. 4E). To ensure that the absence of an effect on ZIKV was not due to the time of sampling, a time course of p400 knockdown and ZIKV infection was performed with samples analyzed at 24, 48, and 72 hpi. *p400* expression was significantly reduced at all time points (Fig. S2A); however, luciferase expression was not changed at any time point assessed (Fig. S2B).

10.1128/mSphere.00081-20.2 FIG S2 *p400* knockdown does not affect ZIKV replication at 24, 48, or 72 h. (A) *p400* knockdown efficiency in Aag2 cells was analyzed by RT-qPCR using the 2^−ΔΔ^*^CT^* method and *p400* expression calculated relative to eGFP dsRNA transfection. Bars show the fold change in gene expression of 3 independent experiments (3 wells in each independent experiment, with an average of three wells per condition/experiment used for statistical analysis). Error bars represent the minimum and maximum fold change. The results were analyzed by a one-sample *t* test. *p400* transcript levels are significantly reduced after dsp400 transfection (*P* = 0.0035, 0.0258, and 0.0057;*, *P* < 0.05; **, *P* < 0.01). (B) The effect of p400 knockdown on ZIKV-NLuc was assessed by luciferase assay. Points show the means of 3 independent experiments (3 wells in each independent experiment, with average of three replicates per condition/experiment used for statistical analysis). Error bars show the standard error of mean. Download FIG S2, TIF file, 0.08 MB. Copyright © 2020 McFarlane et al. 2020 McFarlane et al. This content is distributed under the terms of the Creative Commons Attribution 4.0 International license.

### p400 is required for exo-siRNA pathway activity and regulates levels of *ago-2 in vivo*.

**FIG 5:**
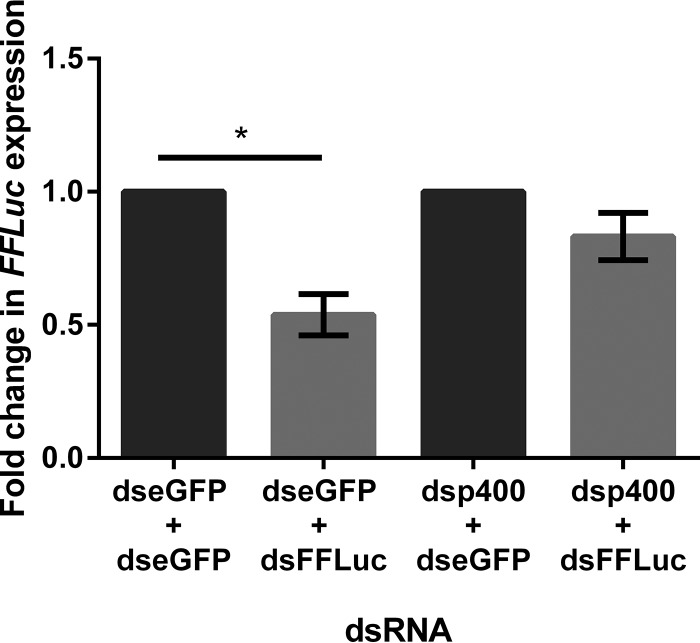
*p400* knockdown leads to reduced silencing efficiency. RNA silencing activity in the presence of *p400* knockdown was determined using a sensor assay. Aag2 cells were transfected with plasmids constitutively expressing firefly (*FFLuc*) or *Renilla* luciferase and dsRNA targeting either p400 or eGFP as a control. At 24 h post-initial transfection, dsRNA against *FFLuc* or eGFP was transfected. Silencing activity was assessed by measuring the relative levels of *FFLuc* 24 h post-second transfection after normalization to *RLuc* (internal transfection control) levels. The level of silencing was calculated relative to respective control eGFP dsRNA-transfected samples (dseGFP+dseGFP as a control for condition dseGFP+dsFFLuc, and dsp400+dseGFP as a control for condition dsp400+dsFFluc), which were set to 1. Bars show the means from 3 independent experiments (3 wells in each independent experiment, average of three wells per condition/experiment used for statistical analysis). Error bars show the standard error of mean. Significance was determined by an unpaired *t* test to determine the *P* value (*P* = 0.034; *, *P* < 0.05).

As p400 had previously been identified as being a component of the exo-siRNA pathway in D. melanogaster-derived S2 cells (42), and we have shown here that it affects arbovirus replication both *in vitro* and *in vivo*, we sought to determine if the antiviral action of p400 could be due to an effect on the exo-siRNA pathway-silencing activity in A. aegypti. In order to determine if p400 was important for exo-siRNA pathway activity, reporter assays were performed. Aag2 cells were transfected with pIZ-FLuc (expressing the silencing target *FFLuc*), pAcIE1-RLuc (as an internal transfection control, expressing Renilla luciferase), and dsp400 or dseGFP (control). At 24 hpt, cells were transfected again with dsRNA targeting either eGFP (control) or *FFLuc* (dsFFluc). Cells were lysed 24 h after the second transfection, and luciferase activity was determined. While the introduction of dsFFLuc in cells treated with dseGFP significantly decreased *FFLuc* expression, silencing of the *FFLuc* reporter plasmid after the introduction of dsFFLuc was abolished in the presence of p400-targeting dsRNA (Fig. 5), showing that p400 is required for the activity of the exo-siRNA pathway.

**FIG 6:**
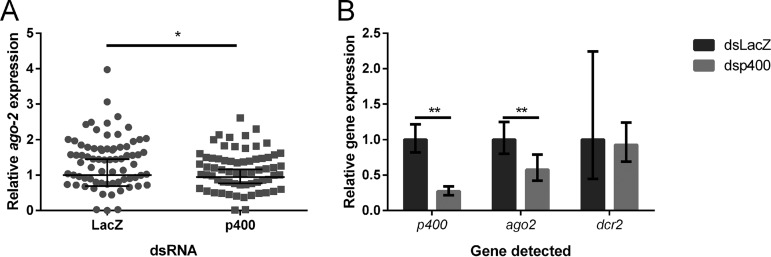
Effect of *p400* knockdown on *ago-2* and *dcr-2* transcript levels in A. aegypti females. (A) *ago-*2 transcript levels in whole dsLacZ- or dsp400-injected females 3 days after an SFV4-infected blood meal was analyzed by RT-qPCR. Normalized expression-per-sample values were obtained as described by Taylor et al. (78) in order to obtain normalized expression values, relative to the ribosomal S7 transcript as a reference, with a geomean of 1 for the dsLacZ control group. Bars show the geomean and 95% confidence intervals from *n* = 73 dsLacZ and *n* = 66 dsp400 females of a single experiment, also used in Fig. 3. This experiment is representative of two independent experiments (second replicate shown in Fig. S1). The results were analyzed using a Mann-Whitney test using log_2_-normalized expression values. *ago-2* transcript levels are significantly reduced in dsp400-injected females compared to those in dsLacZ-injected ones (mean fold change, 1.2; geomean fold change, 1.1; *P* = 0.0197; *, *P* < 0.05). (B) Transcript levels of *p400*, *ago-2,* and *dcr-2* in whole non-blood-fed (NBF) females 4 days after injection with dsLacZ or dsp400 were analyzed by RT-qPCR using the 2^−ΔΔ^*^CT^* method and expression in dsLacZ females as the reference sample. Bars represent the fold change in gene expression (3 independent biological replicates, with pools of 10 females per replicate). Error bars show the minimum and maximum fold change. The results were analyzed using a one-sample *t* test. *p400* and *ago-2* transcript levels are significantly reduced in dsp400-injected females compared to those in dsLacZ-injected females (70% reduction, *P* = 0.0047; and 42% reduction, *P* = 0.0067; **, *P* < 0.01).

Next, we aimed to identify what role p400 might play in the exo-siRNA pathway. In human cells, p400 is part of the Tip60 complex and is involved in transcriptional regulation through chromatin remodeling (43). We therefore reasoned that p400 may play a regulatory role in the exo-siRNA pathway at the transcriptional level. In order to test this hypothesis, we investigated the expression levels of the exo-siRNA effector Ago-2 in SFV4-infected females following the knockdown of p400. The female mosquitoes injected with LacZ (control) or p400-targeting dsRNA and given a blood meal containing SFV4 at 4 days pdi (used in Fig. 3B) were further analyzed for this purpose. RT-qPCR was performed to assess transcript levels in whole females at 3 dpi. As shown in Fig. 6A, *ago-2* transcript expression levels in whole females were significantly reduced upon *p400* transcript knockdown *in vivo*. As described previously, the results were repeated in a second independent experiment (Fig. S1). Similarly, *ago-2* transcript expression levels were also reduced in whole noninfected, NBF females following knockdown of *p400* transcripts (Fig. 6B). In contrast, knockdown of *p400* had no significant effect on *dcr-2* transcript levels.

## DISCUSSION

In this study, we found that p400, an ortholog of the TIP60 complex D. melanogaster protein Domino, is antiviral against the arbovirus SFV in both A. aegypti mosquito-derived cells in culture and adult females. We show that p400 is also antiviral against the related alphavirus CHIKV as well as the bunyavirus BUNV. As already discussed, antiviral activity against WNV (a mosquito-borne flavivirus; *Flavivirida*e) and VSV (*Rhabdoviridae*) has previously been attributed to the p400 ortholog Domino in D. melanogaster-derived DL1 cells following a dsRNA screen for antiviral proteins (38). The knockdown of other members of the Tip60 complex (RuvBL1, RuvBL2, and Tip60) in mosquito cells was also shown to increase WNV and VSV replication (38), suggesting that p400/Tip60 has broad and conserved antiviral activity in dipterans. In mammals, Tip60 has been reported to have antiviral activity against adenovirus (44). In contrast, Tip60 can also promote infections with human papillomavirus (45, 46), human immunodeficiency virus 1 (47, 48), and herpesviruses (46, 49). Therefore, the action of the Tip60 complex, including p400, appears to have a positive or negative effect on virus infection depending on the biological system and virus.

We further show that p400 is required for the activity of the exo-siRNA pathway. Our results are consistent with a previous study in D. melanogaster-derived S2 cells in which Domino was identified as a regulator of the siRNA pathway (42). As the exo-siRNA pathway limits SFV, BUNV, and CHIKV infection in mosquitoes (22, 23, 35), the action of p400 against these viruses is likely to be mediated at least in part through this antiviral pathway. In D. melanogaster, Domino is required for diptericin induction in the fat body (50). Moreover, homozygous *dom* mutant D. melanogaster larvae do not contain hemocytes and do not survive after pupariation, illustrating the important role that Domino has in immunity (51). Roles of this protein also extend to cell proliferation/growth and death (52, 53) and maintaining repression of proapoptotic genes (54). In mammals, Tip60 has been reported to be antiviral by suppressing adenovirus gene expression through binding to the immediate early promoter (44). Considering the different roles of p400 previously described, we cannot therefore exclude that p400 could mediate antiviral effects in A. aegypti mosquitoes through other mechanisms in addition to its role in the exo-siRNA pathway.

Consistent with our finding that p400 regulates *ago-2* transcript levels, p400/Domino is a chromatin-remodeling ATPase member of the Tip60 complex involved in transcriptional regulation and chromatin modification in the fly model but also in human cells (43, 55, 56). In human cells, Tip60/p400 complexes catalyze the incorporation of the histone variant H2A.Z within chromatin, including promoter regions to modulate gene expression in response to diverse cellular cues (57). As Ago-2 is a main effector in the exo-siRNA pathway, the action of p400 on this pathway activity is likely to be mediated through the regulation of *ago-2* expression. This could also be the reason why Domino is required for the Ago-2-dependent RNA interference (RNAi) activity in D. melanogaster-derived S2 cells (42). While p400 regulates *ago-2* transcript levels, p400 knockdown does not have any significant impact on the transcript levels of another important exo-siRNA pathway component, *dcr-2*. Previous reports have shown that Ago-2 is antiviral against SFV, BUNV, and CHIKV (22, 23, 35), while we and others have shown that reduced or absent Ago-2 activity does not result in an increase in ZIKV replication (34, 58, 59). Altogether, this could explain why p400 is antiviral against SFV, BUNV, and CHIKV, while we could not detect any significant effect against ZIKV. As previously discussed, p400 is an ATPase part of the Tip60 chromatin remodeling complex (43, 55, 56). Although the effect of p400 knockdown was not significant on ZIKV, the slight increase in ZIKV expression may indicate that p400 could regulate the expression of other antiviral genes in addition to *ago-2*. Outside antiviral responses, p400 knockdown could even disturb virus replication in other ways, such as changes in the expression of host factors.

We found that *p400* was expressed across various tissues in non-blood-fed females, with the strongest expression in ovaries. In the germarium, the protein is detected in both the germ line and somatic cells, more predominantly in the germ line stem cells and developing secondary follicle. In the primary previtellogenic follicle, p400 is slightly expressed in some somatic epithelial cells. This is similar to the ovarian expression pattern of the two alternative splicing isoforms, DomA and DomB, in D. melanogaster adult females (60), with an expression in the developing cysts within the germarium and little to no expression at stages 7 to 8 (corresponding to the primary follicle arrested at the previtellogenic stage in mosquitoes). In the fruit fly, Domino is required for oogenesis, more particularly for somatic and germ line stem cell maintenance as well as cystocyte differentiation (56, 61, 62). In humans, there are two homologues of Domino, p400 and SRCAP. Interestingly, SRCAP can rescue the female sterility of hypomorphic *dom* alleles in D. melanogaster (63), showing functional conservation of these SWR1-like remodelers during evolution. Due to the similar localization in ovaries of *Aedes* and *Drosophila* spp., it is likely that p400 in mosquitoes and Domino in flies exert similar functions during oogenesis. The protein p400 is also expressed in the large nucleus of the ovarian sheath cells, as well as in the nucleus of the cells forming the crop, suggesting a role in transcriptional regulation in these cells. We also found p400 expressed in tracheae surrounding various tissues, including salivary glands, digestive tract, and ovaries. Interestingly, the von Hippel-Lindau tumor suppressor gene (VHL) regulates the expression of p400 posttranscriptionally in mouse embryonic fibroblasts to prevent senescence (64). In D. melanogaster, VHL regulates tracheal branch migration and lumen formation through endocytosis (65). The presence of p400 in the mosquito tracheal system could indicate that it is involved in tracheal morphogenesis. As arboviruses can infect tracheal cells in A. aegypti (66), p400 could also mediate antiviral protection in these cells. Consistent with its role in hemocyte proliferation and differentiation in the fly model (51), *p400* is expressed in hemocytes in A. aegypti females, with the protein localized in the nucleus of undifferentiated prohemocytes and in the nucleus and cytoplasm of differentiated hemocytes. In D. melanogaster, hemocytes control viral infections by clearing virus-infected cells by phagocytosis (67, 68) and by providing an Ago-2-dependent protection to naive cells (69), resulting in higher viral loads in flies depleted of hemocytes. Consequently, p400 could indirectly control viral loads in A. aegypti due to a role in immune cell differentiation. Hemocytes can be infected by viruses *in vivo* in mosquitoes (27, 70); therefore, the antiviral action of p400 could be directly mediated through Ago-2, though we cannot exclude other mechanisms. In the absence of genetic tools to drive p400 knockdown in a tissue-specific manner in mosquitoes, it is difficult to conclude with certitude *in vivo* in which tissues/cells p400 is antiviral.

The exo-siRNA pathway is one of the key antiviral immune pathways in mosquitoes (8, 16, 19). Despite the importance of this pathway in antiviral defense, there is a considerable lack of understanding regarding how the pathway is regulated. Here, we show a role of p400 in the regulation of *ago-2* transcript levels (and subsequently, the activity of the exo-siRNA pathway) and the antiviral response in A. aegypti. In addition to extending the diverse functions of p400, it could explain the previous observations of Domino’s antiviral activity in D. melanogaster cells and its role in regulating the exo-siRNA pathway. Further studies will need to determine if and how exo-siRNA pathway activity is differentially regulated across tissues in A. aegypti and whether this is relevant for the transmission of arboviruses. In addition, more work will be necessary to understand the mechanism by which p400 is antiviral. This could include interaction studies with other cellular proteins, as well as promoter binding studies to assess the wider mode of action of this protein, which might explain how *ago-2* levels are controlled by p400. The tools used in this study (primers, dsRNA, and antibody) target the four p400 splicing isoforms predicted in A. aegypti genome annotation (AaegL5). As there are two splicing isoforms, DomA and DomB, in D. melanogaster with distinct functions during oogenesis (60), it would be interesting to identify which isoform is involved in the exo-siRNA pathway regulation and antiviral activity in these species. Future work on p400 in A. aegypti will have to investigate these key questions on its mode of action and activity across tissues.

## MATERIALS AND METHODS

### Cell culture.

A. aegypti-derived Aag2 cells (obtained from P. Eggleston, Keele University, UK) were cultured in Leibowitz L-15 medium supplemented with 10% fetal bovine serum (FBS; Gibco), 10% tryptose phosphate broth (TPB), and penicillin-streptomycin. Baby hamster kidney 21 (BHK-21) cells (a commonly used cell line available at the MRC-University of Glasgow Centre for Virus Research) were cultured in Glasgow minimum essential medium (GMEM), 10% newborn calf serum (NBCS) or FBS, 10% TPB, and penicillin-streptomycin. A549 (human lung adenocarcinoma) NPro cells (a gift of R. E. Randall, University of St. Andrews, UK) were cultured in Dulbecco’s modified Eagle’s medium (DMEM) supplemented with 10% FBS and penicillin-streptomycin, and the presence of bovine viral diarrhea virus (BVDV)-derived NPro, which targets interferon regulatory factor 3 (IRF3) for degradation (71), was maintained by the addition of blasticidin (10 μg/ml). Aag2 cells were maintained at 28°C and BHK-21 and A549 NPro cells at 37°C with 5% CO_2_.

### Mosquito rearing.

A. aegypti Liverpool strain (a gift from E. Devaney, University of Glasgow, UK) was reared at 28°C and 80% humidity with a 12:12 light photoperiod. Larvae were reared in water and fed on dry cat food from larvae hatching to the pupal stage. The emerging adult mosquitoes were removed and put in cages with unlimited access to 10% (wt/vol) sucrose solution. For mass rearing, female mosquitoes were fed on heparinized rabbit blood (Orygen Antibodies Ltd.) using a Hemotek system (Hemotek Ltd., UK).

### Reporter virus stocks, plaque assay titration, and infection.

Luciferase expressing SFV4 contains *FFLuc* inserted between nsP3 and nsP4 using duplicated nsP2 cleavage sites, as previously described (25, 72). pCMV-SFV4(3H)-*FFLuc* was electroporated into BHK-21 cells and incubated at 37°C until extensive cell death was visible. The cell supernatant was harvested and clarified by centrifugation. The resulting virus stock was stored at –80°C. CHIKV expressing *FFLuc* between the nonstructural and structural open reading frames under a duplicated subgenomic promoter, CHIKV-2SG-*FFLuc* (25), was rescued by transfection of *in vitro*-transcribed RNA into BHK-21 cells. For this, pSP6-ICRES1-2SG-*FFLuc* was linearized and *in vitro* transcribed using the SP6 MEGAscript kit (Thermo Fisher Scientific), along with Ribo m7G Cap Analog (Promega), followed by transfection into BHK-21 cells using Lipofectamine 2000 (Thermo Fisher Scientific), following the manufacturer’s instructions. The supernatant was collected, clarified by centrifugation, and stored at –80°C. SFV4(3H)-*FFLuc* and CHIKV-2SG-*FFLuc* were titrated on BHK-21 cells by making 10-fold serial dilutions and overlaying the inoculum with 0.6% Avicel in minimum essential medium (MEM) containing 2% FBS and incubating at 37°C for 72 h. Cells were fixed using 10% formalin (Sigma) and stained with 0.1% toluidine blue. NLuc-expressing BUNV was grown and titrated as described previously (35). ZIKV-NLuc (40) was grown on A549 NPro cells cultured in DMEM supplemented with 2% FBS and blasticidin (2 μg/ml) for 5 days at 37°C with 5% CO_2_. Virus supernatant was collected and cleared by centrifugation at 3,220 × *g* for 10 min, followed by storage at –80°C. For virus titration, cell monolayers of A549 NPro were infected with serially diluted ZIKV-NLuc in DMEM with 2% FBS and incubated with an overlay consisting of MEM with 4% FBS, 4% HEPES, and 1.2% Avicel for 5 days. Infected cells were fixed with 4% formaldehyde and stained with 0.2% bromophenol to visualize plaques. Aag2 cells were infected by removing the culture medium, overlaying with 200 μl virus-containing inoculum, and incubating at 28°C for 1 h. The inoculum was removed and replaced with complete medium, followed by incubation at 28°C.

Aag2 cell infections were carried out with CHIKV-2SG-*FFLuc* at a multiplicity of infection (MOI) of 0.02, with SFV4(3H)-*FFLuc* at an MOI of 0.1, with BUNV-NLuc at an MOI of 0.01, or with ZIKV-NLuc at an MOI of 0.01 24 h posttransfection (hpt). SFV- and CHIKV-infected cells were lysed at 24 hpi, BUNV-infected cells were lysed at 48 hpi, and ZIKV-infected cells were lysed at 72 hpi, and luciferase activity was measured.

### SFV production for oral infection of mosquitoes.

SFV4 was produced from plasmid pCMV-SFV4, as described previously (73). Briefly, the plasmid was transfected using Lipofectamine 2000 (Thermo Fisher Scientific) into BHK-21 cells grown in GMEM with 2% FBS and 10 % TPB at 37°C with 5 % CO_2_. The virus was titrated by a plaque assay on BHK-21 cells, as described above.

### Plasmids.

The pIZ-FLuc and pAcIE1-RLuc plasmids have been previously described (74, 75). The plasmid pPUb-V5MBP was synthesized by subcloning maltose-binding protein (MBP) from pcDNA-DEST40-MBP-hDVR (75) into the mosquito expression vector pPUb (29), based on an expression construct containing the A. aegypti polyubiquitin promoter (76). The plasmid pUC57-p400 was synthesized (GenScript) and contains the A. aegypti coding sequence, as indicated in VectorBase with accession number AAEL001440 (assembly AaegL1). In the AaegL5 assembly, AAEL001440 was changed to AAEL027494 (transcript identifiers [IDs] RA to RD; all transcripts targeted by dsp400). The p400 coding sequence was further subcloned into the pPUb expression vector. The V5 tag was added to the N terminus of pPUb-MBP and pPUb-p400 clones using the Infusion cloning technique (Clontech) to give pPUb-V5MBP and pPUb-V5p400, respectively.

### dsRNA synthesis for *in vitro* experiments.

RNA was extracted from Aag2 cells using TRIzol (Thermo Fisher Scientific), following the manufacturer’s guidelines. One microgram of RNA was reverse transcribed using SuperScript III (Thermo Fisher Scientific) and an oligo(dT)15 primer (Promega), according to the manufacturer’s instructions. Unique portions of candidate genes were amplified from cDNA with primers containing T7 RNA polymerase minimal promoter sequence overhangs using GoTaq G2 Flexi polymerase (Promega). PCR products were sequenced for target verification before the production of dsRNA. For the production of dseGFP (used as a control), specific primers with T7 RNA polymerase promoter sequences were used to amplify a unique portion of eGFP from a plasmid template containing the eGFP-encoding gene. All primer sequences are listed in Table S1. The MEGAscript RNAi kit (Thermo Fisher Scientific) was used to synthesize dsRNA from the PCR fragments, according to the manufacturer’s guidelines.

10.1128/mSphere.00081-20.5 TABLE S1 Primers used for dsRNA synthesis and RT-qPCR to check knockdown efficiency or analyze transcript levels. The sequence marked in bold is T7 minimal promoter sequence. Download Table S1, DOCX file, 0.02 MB. Copyright © 2020 McFarlane et al. 2020 McFarlane et al. This content is distributed under the terms of the Creative Commons Attribution 4.0 International license.

### dsRNA synthesis and purification for *in vivo* experiments.

Total RNA was extracted with TRIzol (Thermo Fisher Scientific) from whole NBF A. aegypti females, according to the manufacturer’s instructions, including DNase treatment (Turbo DNase; Ambion). cDNA was generated from 1 μg of total RNA using SuperScript III reverse transcriptase (Thermo Fisher Scientific) and oligo(dT)15. cDNAs were further amplified with KOD Hot Start master mix (EMD Millipore). p400-specific primers (Table S1) with a T7 RNA polymerase promoter sequence were used to amplify a p400-derived fragment (same primers used for *in vitro* and *in vivo* experiments) and further purified using the QIAquick gel extraction kit (Qiagen). After sequencing, the PCR product was used as the template for a second PCR using the same primers and polymerase. For the production of dsLacZ (used as control dsRNA), specific primers with T7 RNA polymerase promoter sequences were used to amplify a *lacZ*-derived fragment from plasmid template *Drosophila/*act5C-βGal (stock number 1220 obtained from DGRC) containing the Escherichia coli *lacZ* gene. dsRNAs were synthesized and purified using the MEGAscript RNAi kit (Thermo Fisher Scientific), according to the manufacturer’s instructions. dsRNA was then purified and concentrated to 10 μg/μl in nuclease-free water using sodium acetate (3 M; Ambion) and ethanol precipitation.

### Transfection of BHK-21 cells.

BHK-21 cells were plated at a density of 3 × 10^6^ cells per well in 6-well plates. One microgram of pPUb-V5MBP or pPUb-V5p400 was transfected the following day using Dharmafect2 (Dharmacon), following the recommended protocol. Cells were collected 24 hpt by trypsinization and plated in slides (ibidi) for immunostaining.

### Transfection of mosquito cells.

Aag2 cells were plated at a density of 1.7 × 10^5^ cells per well in a 24-well plate. dsRNA or plasmid DNA was transfected the following day with Lipofectamine 2000 (Thermo Fisher Scientific), using the supplied protocol. The medium was replaced at 4 to 5 hpt. Knockdown experiments were performed by transfecting 100 ng dsRNA into Aag2 cells. Plasmid sensor assays were performed by transfecting 50 ng pIZ-FLuc and 8 ng pAcIE1-RLuc and 100 ng of either eGFP-targeting or p400-targeting dsRNA. At 24 h post-initial transfection, cells were transfected again with 10 ng FFLuc- or eGFP-targeting dsRNA. The *FFLuc* and *RLuc* activities were measured at 24 h post-second transfection.

### Luciferase assay.

Aag2 cells were lysed in 1× passive lysis buffer (Promega) and lysed by rocking at room temperature for 20 min or stored in –20°C immediately. *FFLuc* and *Renilla* luciferase activities were measured using the dual-luciferase assay system (or Steady-Glo luciferase assay system in the case of CHIKV-2SG-*FFLuc*) (Promega); NLuc activities were measured by using the Nano-Glo luciferase assay systems (Promega) (ZIKV-NLuc and BUNV-NLuc). All measurements were carried out on Glomax luminometers (Promega).

### Total RNA extraction and RT-qPCR from cells.

For analysis of *p400* knockdown efficiency, cells were lysed in TRIzol reagent (Thermo Fisher Scientific) at 24 hpt and extracted according to the manufacturer’s instructions. Reverse transcription (RT) was performed using the SuperScript III enzyme kit (Thermo Fisher Scientific) from 1 μg of RNA in a final volume of 20 μl. Quantitative PCR (qPCR) was carried out using Fast SYBR green master mix (Applied Biosystems) on a 7500 Fast machine (Applied Biosystems). The primers used are listed in Table S1. Data were analyzed with the 7500 Software v2.0.6, and transcript expression relative to the ribosomal S7 as a reference was calculated according to the 2^−ΔΔ^*^CT^* method (77). The dseGFP control was set to 1, and dsp400 samples were normalized to dseGFP. Data from 3 independent biological replicates were analyzed using a one-sample *t* test (Prism software).

### dsRNA injection into mosquitoes.

At 1 to 2 days after emergence, cold-anesthetized female mosquitoes were injected with dsRNA into their thorax using a nanoinjector (Nanoject II; Drummond Scientific) with 2 μg of dsRNA (dsp400 or dsLacZ) in 414 nl of injection solution. To increase knockdown efficiencies, Cellfectin II transfection reagent (Thermo Fisher Scientific) was added to the injection solution. Briefly, Cellfectin was mixed with Schneider’s *Drosophila* medium (1:1 [vol/vol]; Thermo Fisher Scientific); this mix was added to dsRNA solution (1:1 [vol/vol]) previously adjusted with Schneider’s medium to give 2 μg of dsRNA per female/injection volume. The injection solution was then incubated for 15 min at room temperature before injection.

### Mosquito sampling, dissection, and hemocyte perfusion.

To assess the effect of p400 knockdown on *ago-2* transcript levels in whole NBF females and in tissues, female mosquitoes injected with dsRNA (targeting control or p400 transcripts) were sacrificed at 4 days pdi. Whole females (pools of 10 females) and dissected tissues (pools of 25 digestive tracts or ovaries, pools of 60 salivary glands, and pools of perfused hemocytes from 70 females) were further stored in tubes on dry ice before TRIzol was added for RNA extraction. Digestive tracts include the posterior part of the esophagus, dorsal diverticula, crop, midgut, Malpighian tubules, and hindgut. The tissues were carefully dissected from 5-day-old NBF females in RNase-free 0.05% (vol/vol) phosphate-buffered saline with Tween 20 (PBS-T) and either placed into tubes on dry ice before TRIzol was added for subsequent analysis of *p400* and *ago-2* tissue levels by qPCR or added into 0.05% PBS-T on wet ice for fixation and immunostaining. Hemocytes were collected by perfusion. Briefly, the last segment of the abdomen was cut, and mosquitoes were then injected in the thorax with 0.01% PBS-T using a glass capillary mounted on a syringe. For RT-qPCR experiments, PBS-T-diluted hemocytes were collected in tubes on ice and centrifuged at 375 × *g* for 15 min at 4°C. The supernatant was gently removed before adding TRIzol. For immunostaining, hemocytes were perfused on slides (ibidi; 5 females per well, 4 wells per replicate). The slides were left for 30 min at 28°C before removal of PBS-T and fixation.

### Antibody production.

The Peptide Supplied Polyclonal Antibody Package (mouse; catalog no. SC1046) available from GenScript USA, Inc. was used to produce the p400 antibody. Peptide optimization was performed using the OptimumAntigen design program provided by GenScript. The peptide GMNKPKAIQDQNTSC (present in proteins AAEL027494-RA to AAEL027494-RD) was selected and conjugated to keyhole limpet hemocyanin (KLH) for immunization into five BALB/c mice. Antiserum from mouse 5 was used in these experiments. The specificity of the antibody was confirmed by colocalization of p400 and V5 antibody signals in BHK-21 cells transfected with pPUb-V5p400 (Fig. S3). Further details on antibody production are available upon request.

10.1128/mSphere.00081-20.3 FIG S3 Expression and detection of V5-tagged p400 or MBP in BHK-21 cells. The expression of p400 and V5 was analyzed in BHK-21 cells transfected with pPUb-V5p400 or pPUb-V5MBP plasmids, encoding V5-tagged p400 (A to C) or V5-tagged MBP (D to F) by an immunofluorescence assay using anti-p400 antibody and anti-V5 antibody. Signals were determined using an Alexa Fluor 568 goat anti-mouse IgG (H + L) (red, p400) and an Alexa Fluor 488 goat anti-rabbit IgG (H + L) (green, V5). Panels C and F show merged images of A and B and of D and E, respectively. Nuclei are stained by DAPI (blue) and F-actin by phalloidin 647 (white). Images were acquired on a Zeiss LSM 710 inverted confocal microscope with a 63× oil immersion objective. Scale bar = 20 μm. Download FIG S3, TIF file, 2.7 MB. Copyright © 2020 McFarlane et al. 2020 McFarlane et al. This content is distributed under the terms of the Creative Commons Attribution 4.0 International license.

### Immunofluorescence analysis of mosquito tissues, hemocytes, and BHK-21 cells.

Salivary glands and ovaries (minimum of *n* = 10 each for 1 experiment for 3 independent experiments) were fixed at room temperature (RT) for 20 min in 4% (w/vol) paraformaldehyde (PFA) diluted in 0.05% PBS-T. BHK-21 cells (2 independent experiments), perfused hemocytes, and digestive tract tissues (*n* = 10 minimum for 1 experiment for 3 independent experiments) were fixed in the same way, except that PFA was diluted using PBS. Fixed cells/tissues (except ovaries) were washed (three times for 15 min each at 4°C) in 0.05% PBS-T before blocking for at least 30 min in blocking solution (0.05% PBS-T, 5% [vol/vol] fetal calf serum [FCS], 5% [wt/vol] bovine serum albumin [BSA], 0.05% [vol/vol] Triton X-100) at 4°C. Unlike other tissues, ovaries were dilacerated and washed in 1% PBS-T before being blocked in blocking solution containing 0.5% (vol/vol) Tween and 0.5% (vol/vol) Triton X-100. Cells/tissues were incubated at 4°C overnight with a mouse anti-p400 antibody (see below) diluted 1:200 or a rabbit anti-V5 (Abcam) at 1:500 in blocking solution. For each experiment, a negative control without primary antibody was carried out (Fig. S4). Samples were washed (five times for 15 min each at 4°C) in 0.05% PBS-T (0.5% PBS-T for ovaries) and incubated with an Alexa Fluor 568 goat anti-mouse IgG (H+L) or an Alexa Fluor 488 goat anti-rabbit IgG (H+L) diluted 1:1,000, 1× 4′,6-diamidino-2-phenylindole (DAPI) (405 nm), and 1× phalloidin (488 or 647 nm) in blocking solution for 2 h at RT. Four washes in 0.05% PBS-T (0.5% PBS-T for ovaries) were carried out. Tissues were mounted between the slide and coverslip (24 mm by 24 mm) with an imaging spacer (1 well, 13-mm diameter, 0.12-mm thickness, Grace Bio-Labs SecureSeal imaging spacer; Sigma-Aldrich) using mounting medium (ibidi). Mounting medium was used to replace PBS-T in hemocyte- or BHK-21-containing wells. Images were acquired on a Zeiss LSM 710 inverted confocal microscope, equipped with a 40×, 63×, or 100× oil immersion objective, and processed with Fiji/ImageJ and Adobe Photoshop.

10.1128/mSphere.00081-20.4 FIG S4 Immunofluorescence assay in the absence of primary antibody in tissues of NBF A. aegypti females. Immunofluorescence assay without p400 primary antibody on perfused hemocytes (A and B), salivary glands (C), crop (D), midgut (E), and ovaries (F). The signal was revealed by using an Alexa Fluor 568 goat anti-mouse IgG (H+L) (red); nuclei are stained by DAPI (blue), and F-actin is stained by phalloidin 488 (green). Images were acquired on a Zeiss LSM 710 inverted confocal microscope with a 40× or 63× oil immersion objective and using the same parameters as for the samples incubated with p400 primary antibody. Scale bars are 10 μm (A and B) and 40 μm (C to F). Download FIG S4, TIF file, 1.0 MB. Copyright © 2020 McFarlane et al. 2020 McFarlane et al. This content is distributed under the terms of the Creative Commons Attribution 4.0 International license.

### Blood meal infection of mosquitoes with SFV.

At 4 days pdi, A. aegypti females were allowed to feed for 30 min on blood meal containing SFV4. Fresh rabbit blood (Orygen Antibodies Ltd.) was washed with PBS to remove white blood cells and serum. PBS was then added to the red blood cell fraction to return to the initial blood volume. The infectious blood meal was prepared with 2/3 of washed blood and 1/3 of SFV-containing culture medium to give a final titer of 5.10^7^ PFU/ml, supplemented with 2 mM ATP. Only engorged females were kept further in the presence of 10% sucrose at 28°C and 80% humidity. At 3 days postfeeding, females were sampled individually before RNA extraction.

### Total RNA extraction and RT-qPCR from mosquitoes.

Dissected tissues or whole females were homogenized in TRIzol reagent with glass beads using the Precellys 24 homogenizer (Bertin Instruments). RNA from tissues, whole females, and hemocytes was extracted according to the manufacturer’s instructions, except that 1-bromo-3-chloropropane was used instead of chloroform, and DNase (Turbo DNase; Ambion) treatment was carried out. Reverse transcription (RT) was performed using the Moloney murine leukemia virus (MMLV) retrotranscriptase (Promega) from 1 to 2 μg of RNA in a final volume of 40 μl. cDNA was aliquoted and further stored at –20°C until qPCR was carried out using Fast SYBR green master mix (Applied Biosystems) on a 7500 Fast machine (Applied Biosystems). The primers used are listed in Table S1. Data were analyzed with the 7500 Software v2.0.6.

For analysis of *p400* expression, transcript expression relative to the ribosomal S7 transcript as a reference was calculated according to the 2^−ΔΔ^*^CT^* method (77). The hemocyte sample was set to 1, and other samples were normalized to the hemocyte sample. Data were obtained from 3 independent biological replicates. For analysis of *p400* knockdown efficiency and the effect on *ago-2* levels in whole NBF females, transcript expression relative to the ribosomal S7 transcript as a reference was calculated according to the 2^−ΔΔ^*^CT^* method (77). The dsLacZ control was set to 1, and dsp400 samples were normalized to dsLacZ. Data from 3 independent biological replicates (pool of 10 females per replicate) were analyzed by a one-sample *t* test (Prism software). For analysis of *p400* knockdown and the effect on *ago-2* levels and SFV infection, data were analyzed as described by Taylor et al. (78) in order to obtain normalized expression values, relative to the ribosomal S7 transcript as a reference, with a geometric mean (geomean) of 1 for the dsLacZ control group (two independent experiments; *n* = 73 females for dsLacZ and 66 for dsp400 in Fig. 3A and B and 6A; *n* = 39 females for dsLacZ and 43 for dsp400 in Fig. S1). Log_2_-normalized expression values were analyzed using a Mann-Whitney test (with the Prism software).

### Data availability.

The data sets generated and analyzed during the current study are available in the University of Glasgow repository at https://doi.org/10.5525/gla.researchdata.705.

## References

[1] Weaver SC, Reisen WK 2010 Present and future arboviral threats. Antiviral Res 85:328–345. doi:10.1016/j.antiviral.2009.10.008. 19857523 PMC2815176

[2] Weaver SC, Costa F, Garcia-Blanco MA, Ko AI, Ribeiro GS, Saade G, Shi PY, Vasilakis N 2016 Zika virus: history, emergence, biology, and prospects for control. Antiviral Res 130:69–80. doi:10.1016/j.antiviral.2016.03.010. 26996139 PMC4851879

[3] Linthicum KJ, Britch SC, Anyamba A 2016 Rift Valley fever: an emerging mosquito-borne disease. Annu Rev Entomol 61:395–415. doi:10.1146/annurev-ento-010715-023819. 26982443

[4] Mayer SV, Tesh RB, Vasilakis N 2017 The emergence of arthropod-borne viral diseases: a global prospective on dengue, chikungunya and Zika fevers. Acta Trop 166:155–163. doi:10.1016/j.actatropica.2016.11.020. 27876643 PMC5203945

[5] Paixão ES, Teixeira MG, Rodrigues LC 2018 Zika, chikungunya and dengue: the causes and threats of new and re-emerging arboviral diseases. BMJ Glob Health 3:e000530. doi:10.1136/bmjgh-2017-000530. PMC5759716 29435366

[6] Wilder-Smith A, Gubler DJ, Weaver SC, Monath TP, Heymann DL, Scott TW 2017 Epidemic arboviral diseases: priorities for research and public health. Lancet Infect Dis 17:e101–e106. doi:10.1016/S1473-3099(16)30518-7. 28011234

[7] Kean J, Rainey SM, McFarlane M, Donald CL, Schnettler E, Kohl A, Pondeville E 2015 Fighting arbovirus transmission: natural and engineered control of vector competence in *Aedes* mosquitoes. Insects 6:236–278. doi:10.3390/insects6010236. 26463078 PMC4553541

[8] Blair CD, Olson KE 2015 The role of RNA interference (RNAi) in arbovirus-vector interactions. Viruses 7:820–843. doi:10.3390/v7020820. 25690800 PMC4353918

[9] Johnson KN 2015 The impact of *Wolbachia* on virus infection in mosquitoes. Viruses 7:5705–5717. doi:10.3390/v7112903. 26556361 PMC4664976

[10] Alphey L 2014 Genetic control of mosquitoes. Annu Rev Entomol 59:205–224. doi:10.1146/annurev-ento-011613-162002. 24160434

[11] Lindsey ARI, Bhattacharya T, Newton ILG, Hardy RW 2018 Conflict in the intracellular lives of endosymbionts and viruses: a mechanistic look at *Wolbachia*-mediated pathogen-blocking. Viruses 10:141. doi:10.3390/v10040141. PMC5923435 29561780

[12] Terradas G, McGraw EA 2017 *Wolbachia*-mediated virus blocking in the mosquito vector Aedes aegypti. Curr Opin Insect Sci 22:37–44. doi:10.1016/j.cois.2017.05.005. 28805637

[13] Jiggins FM 2017 The spread of *Wolbachia* through mosquito populations. PLoS Biol 15:e2002780. doi:10.1371/journal.pbio.2002780. 28570608 PMC5453404

[14] Macias VM, Ohm JR, Rasgon JL 2017 Gene drive for mosquito control: where did it come from and where are we headed? Int J Environ Res Public Health 14:1006. doi:10.3390/ijerph14091006. PMC5615543 28869513

[15] Adelman ZN, Tu Z 2016 Control of mosquito-borne infectious diseases: sex and gene drive. Trends Parasitol 32:219–229. doi:10.1016/j.pt.2015.12.003. 26897660 PMC4767671

[16] Olson KE, Blair CD 2015 Arbovirus-mosquito interactions: RNAi pathway. Curr Opin Virol 15:119–126. doi:10.1016/j.coviro.2015.10.001. 26629932 PMC4765169

[17] Donald CL, Kohl A, Schnettler E 2012 New insights into control of arbovirus replication and spread by insect RNA interference pathways. Insects 3:511–531. doi:10.3390/insects3020511. 26466541 PMC4553608

[18] Bronkhorst AW, van Rij RP 2014 The long and short of antiviral defense: small RNA-based immunity in insects. Curr Opin Virol 7:19–28. doi:10.1016/j.coviro.2014.03.010. 24732439

[19] Samuel GH, Adelman ZN, Myles KM 2018 Antiviral immunity and virus-mediated antagonism in disease vector mosquitoes. Trends Microbiol 26:447–461. doi:10.1016/j.tim.2017.12.005. 29395729 PMC5910197

[20] Keene KM, Foy BD, Sanchez-Vargas I, Beaty BJ, Blair CD, Olson KE 2004 RNA interference acts as a natural antiviral response to O’nyong-nyong virus (Alphavirus; Togaviridae) infection of *Anopheles gambiae*. Proc Natl Acad Sci U S A 101:17240–17245. doi:10.1073/pnas.0406983101. 15583140 PMC535383

[21] Myles KM, Wiley MR, Morazzani EM, Adelman ZN 2008 Alphavirus-derived small RNAs modulate pathogenesis in disease vector mosquitoes. Proc Natl Acad Sci U S A 105:19938–19943. doi:10.1073/pnas.0803408105. 19047642 PMC2604946

[22] Schnettler E, Donald CL, Human S, Watson M, Siu RW, McFarlane M, Fazakerley JK, Kohl A, Fragkoudis R 2013 Knockdown of piRNA pathway proteins results in enhanced Semliki Forest virus production in mosquito cells. J Gen Virol 94:1680–1689. doi:10.1099/vir.0.053850-0. 23559478 PMC3709635

[23] McFarlane M, Arias-Goeta C, Martin E, O'Hara Z, Lulla A, Mousson L, Rainey SM, Misbah S, Schnettler E, Donald CL, Merits A, Kohl A, Failloux A-B 2014 Characterization of *Aedes aegypti* innate-immune pathways that limit chikungunya virus replication. PLoS Negl Trop Dis 8:e2994. doi:10.1371/journal.pntd.0002994. 25058001 PMC4109886

[24] Campbell CL, Keene KM, Brackney DE, Olson KE, Blair CD, Wilusz J, Foy BD 2008 *Aedes aegypti* uses RNA interference in defense against Sindbis virus infection. BMC Microbiol 8:47. doi:10.1186/1471-2180-8-47. 18366655 PMC2278134

[25] Varjak M, Dietrich I, Sreenu VB, Till BE, Merits A, Kohl A, Schnettler E 2018 Spindle-E acts antivirally against alphaviruses in mosquito cells. Viruses 10:88. doi:10.3390/v10020088. PMC5850395 29463033

[26] Morazzani EM, Wiley MR, Murreddu MG, Adelman ZN, Myles KM 2012 Production of virus-derived ping-pong-dependent piRNA-like small RNAs in the mosquito soma. PLoS Pathog 8:e1002470. doi:10.1371/journal.ppat.1002470. 22241995 PMC3252369

[27] Carissimo G, Pondeville E, McFarlane M, Dietrich I, Mitri C, Bischoff E, Antoniewski C, Bourgouin C, Failloux AB, Kohl A, Vernick KD 2015 Antiviral immunity of *Anopheles gambiae* is highly compartmentalized, with distinct roles for RNA interference and gut microbiota. Proc Natl Acad Sci U S A 112:E176–E185. doi:10.1073/pnas.1412984112. 25548172 PMC4299212

[28] Waldock J, Olson KE, Christophides GK 2012 *Anopheles gambiae* antiviral immune response to systemic O’nyong-nyong infection. PLoS Negl Trop Dis 6:e1565. doi:10.1371/journal.pntd.0001565. 22428080 PMC3302841

[29] Varjak M, Maringer K, Watson M, Sreenu VB, Fredericks AC, Pondeville E, Donald CL, Sterk J, Kean J, Vazeille M, Failloux AB, Kohl A, Schnettler E 2017 *Aedes aegypti* Piwi4 is a noncanonical PIWI protein involved in antiviral responses. mSphere 2:e00144-17. doi:10.1128/mSphere.00144-17. 28497119 PMC5415634

[30] Sánchez-Vargas I, Scott JC, Poole-Smith BK, Franz AW, Barbosa-Solomieu V, Wilusz J, Olson KE, Blair CD 2009 Dengue virus type 2 infections of *Aedes aegypt*i are modulated by the mosquito’s RNA interference pathway. PLoS Pathog 5:e1000299. doi:10.1371/journal.ppat.1000299. 19214215 PMC2633610

[31] Samuel GH, Wiley MR, Badawi A, Adelman ZN, Myles KM 2016 Yellow fever virus capsid protein is a potent suppressor of RNA silencing that binds double-stranded RNA. Proc Natl Acad Sci U S A 113:13863–13868. doi:10.1073/pnas.1600544113. 27849599 PMC5137771

[32] Scott JC, Brackney DE, Campbell CL, Bondu-Hawkins V, Hjelle B, Ebel GD, Olson KE, Blair CD 2010 Comparison of dengue virus type 2-specific small RNAs from RNA interference-competent and -incompetent mosquito cells. PLoS Negl Trop Dis 4:e848. doi:10.1371/journal.pntd.0000848. 21049014 PMC2964303

[33] Franz AW, Sanchez-Vargas I, Adelman ZN, Blair CD, Beaty BJ, James AA, Olson KE 2006 Engineering RNA interference-based resistance to dengue virus type 2 in genetically modified *Aedes aegypti*. Proc Natl Acad Sci U S A 103:4198–4203. doi:10.1073/pnas.0600479103. 16537508 PMC1449670

[34] Varjak M, Donald CL, Mottram TJ, Sreenu VB, Merits A, Maringer K, Schnettler E, Kohl A 2017 Characterization of the Zika virus induced small RNA response in *Aedes aegypti* cells. PLoS Negl Trop Dis 11:e0006010. doi:10.1371/journal.pntd.0006010. 29040304 PMC5667879

[35] Dietrich I, Shi X, McFarlane M, Watson M, Blomstrom AL, Skelton JK, Kohl A, Elliott RM, Schnettler E 2017 The antiviral RNAi response in vector and non-vector cells against orthobunyaviruses. PLoS Negl Trop Dis 11:e0005272. doi:10.1371/journal.pntd.0005272. 28060823 PMC5245901

[36] Dietrich I, Jansen S, Fall G, Lorenzen S, Rudolf M, Huber K, Heitmann A, Schicht S, Ndiaye EH, Watson M, Castelli I, Brennan B, Elliott RM, Diallo M, Sall AA, Failloux AB, Schnettler E, Kohl A, Becker SC 2017 RNA interference restricts Rift Valley fever virus in multiple insect systems. mSphere 2:e00090-17. doi:10.1128/mSphere.00090-17. 28497117 PMC5415632

[37] Léger P, Lara E, Jagla B, Sismeiro O, Mansuroglu Z, Coppee JY, Bonnefoy E, Bouloy M 2013 Dicer-2- and Piwi-mediated RNA interference in Rift Valley fever virus-infected mosquito cells. J Virol 87:1631–1648. doi:10.1128/JVI.02795-12. 23175368 PMC3554164

[38] Yasunaga A, Hanna SL, Li J, Cho H, Rose PP, Spiridigliozzi A, Gold B, Diamond MS, Cherry S 2014 Genome-wide RNAi screen identifies broadly-acting host factors that inhibit arbovirus infection. PLoS Pathog 10:e1003914. doi:10.1371/journal.ppat.1003914. 24550726 PMC3923753

[39] Castillo JC, Robertson AE, Strand MR 2006 Characterization of hemocytes from the mosquitoes *Anopheles gambiae* and *Aedes aegypti*. Insect Biochem Mol Biol 36:891–903. doi:10.1016/j.ibmb.2006.08.010. 17098164 PMC2757042

[40] Mutso M, Saul S, Rausalu K, Susova O, Zusinaite E, Mahalingam S, Merits A 2017 Reverse genetic system, genetically stable reporter viruses and packaged subgenomic replicon based on a Brazilian Zika virus isolate. J Gen Virol 98:2712–2724. doi:10.1099/jgv.0.000938. 29022864

[41] Göertz GP, Vogels CBF, Geertsema C, Koenraadt CJM, Pijlman GP 2017 Mosquito co-infection with Zika and chikungunya virus allows simultaneous transmission without affecting vector competence of *Aedes aegypti*. PLoS Negl Trop Dis 11:e0005654. doi:10.1371/journal.pntd.0005654. 28570693 PMC5469501

[42] Zhou R, Hotta I, Denli AM, Hong P, Perrimon N, Hannon GJ 2008 Comparative analysis of Argonaute-dependent small RNA pathways in *Drosophila*. Mol Cell 32:592–599. doi:10.1016/j.molcel.2008.10.018. 19026789 PMC2615197

[43] Jha S, Dutta A 2009 RVB1/RVB2: running rings around molecular biology. Mol Cell 34:521–533. doi:10.1016/j.molcel.2009.05.016. 19524533 PMC2733251

[44] Gupta A, Jha S, Engel DA, Ornelles DA, Dutta A 2013 Tip60 degradation by adenovirus relieves transcriptional repression of viral transcriptional activator EIA. Oncogene 32:5017–5025. doi:10.1038/onc.2012.534. 23178490 PMC3955737

[45] Smith JA, Haberstroh FS, White EA, Livingston DM, DeCaprio JA, Howley PM 2014 SMCX and components of the TIP60 complex contribute to E2 regulation of the HPV E6/E7 promoter. Virology 468–470:311–321. doi:10.1016/j.virol.2014.08.022. PMC4252969 25222147

[46] Hong S, Dutta A, Laimins LA 2015 The acetyltransferase Tip60 is a critical regulator of the differentiation-dependent amplification of human papillomaviruses. J Virol 89:4668–4675. doi:10.1128/JVI.03455-14. 25673709 PMC4442364

[47] Col E, Caron C, Chable-Bessia C, Legube G, Gazzeri S, Komatsu Y, Yoshida M, Benkirane M, Trouche D, Khochbin S 2005 HIV-1 Tat targets Tip60 to impair the apoptotic cell response to genotoxic stresses. EMBO J 24:2634–2645. doi:10.1038/sj.emboj.7600734. 16001085 PMC1176461

[48] Zhang SM, Song M, Yang TY, Fan R, Liu XD, Zhou PK 2012 HIV-1 Tat impairs cell cycle control by targeting the Tip60, Plk1 and cyclin B1 ternary complex. Cell Cycle 11:1217–1234. doi:10.4161/cc.11.6.19664. 22391203

[49] Li R, Zhu J, Xie Z, Liao G, Liu J, Chen MR, Hu S, Woodard C, Lin J, Taverna SD, Desai P, Ambinder RF, Hayward GS, Qian J, Zhu H, Hayward SD 2011 Conserved herpesvirus kinases target the DNA damage response pathway and TIP60 histone acetyltransferase to promote virus replication. Cell Host Microbe 10:390–400. doi:10.1016/j.chom.2011.08.013. 22018239 PMC3253558

[50] Foley E, O'Farrell PH 2003 Nitric oxide contributes to induction of innate immune responses to gram-negative bacteria in *Drosophila*. Genes Dev 17:115–125. doi:10.1101/gad.1018503. 12514104 PMC195964

[51] Braun A, Lemaitre B, Lanot R, Zachary D, Meister M 1997 *Drosophila* immunity: analysis of larval hemocytes by P-element-mediated enhancer trap. Genetics 147:623–634. 9335599 10.1093/genetics/147.2.623 PMC1208184

[52] Ellis K, Friedman C, Yedvobnick B 2015 *Drosophila* domino exhibits genetic interactions with a wide spectrum of chromatin protein-encoding loci. PLoS One 10:e0142635. doi:10.1371/journal.pone.0142635. 26555684 PMC4640824

[53] Kwon MH, Callaway H, Zhong J, Yedvobnick B 2013 A targeted genetic modifier screen links the SWI2/SNF2 protein domino to growth and autophagy genes in *Drosophila melanogaster*. G3 (Bethesda) 3:815–825. doi:10.1534/g3.112.005496. 23550128 PMC3656729

[54] Ellis K, Wardwell-Ozgo J, Moberg KH, Yedvobnick B 2018 The domino SWI2/SNF2 gene product represses cell death in *Drosophila melanogaster*. G3 (Bethesda) 8:2355–2360. doi:10.1534/g3.118.200228. 29752350 PMC6027882

[55] Kusch T, Florens L, Macdonald WH, Swanson SK, Glaser RL, Yates JR III, Abmayr SM, Washburn MP, Workman JL 2004 Acetylation by Tip60 is required for selective histone variant exchange at DNA lesions. Science 306:2084–2087. doi:10.1126/science.1103455. 15528408

[56] Ruhf ML, Braun A, Papoulas O, Tamkun JW, Randsholt N, Meister M 2001 The domino gene of *Drosophila* encodes novel members of the SWI2/SNF2 family of DNA-dependent ATPases, which contribute to the silencing of homeotic genes. Development 128:1429–1441. 11262242 10.1242/dev.128.8.1429

[57] Subramanian V, Fields PA, Boyer LA 2015 H2A.Z: a molecular rheostat for transcriptional control. F1000Prime Rep 7:01. doi:10.12703/P7-01. 25705384 PMC4311278

[58] Harsh S, Ozakman Y, Kitchen SM, Paquin-Proulx D, Nixon DF, Eleftherianos I 2018 Dicer-2 regulates resistance and maintains homeostasis against Zika virus infection in *Drosophila*. J Immunol 201:3058–3072. doi:10.4049/jimmunol.1800597. 30305326 PMC6219897

[59] Liu Y, Gordesky-Gold B, Leney-Greene M, Weinbren NL, Tudor M, Cherry S 2018 Inflammation-induced, STING-dependent autophagy restricts Zika virus infection in the *Drosophila* brain. Cell Host Microbe 24:57–68.e3. doi:10.1016/j.chom.2018.05.022. 29934091 PMC6173519

[60] Börner K, Becker PB 2016 Splice variants of the SWR1-type nucleosome remodeling factor Domino have distinct functions during *Drosophila melanogaster* oogenesis. Development 143:3154–3167. doi:10.1242/dev.139634. 27578180

[61] Yan D, Neumuller RA, Buckner M, Ayers K, Li H, Hu Y, Yang-Zhou D, Pan L, Wang X, Kelley C, Vinayagam A, Binari R, Randklev S, Perkins LA, Xie T, Cooley L, Perrimon N 2014 A regulatory network of *Drosophila* germline stem cell self-renewal. Dev Cell 28:459–473. doi:10.1016/j.devcel.2014.01.020. 24576427 PMC3998650

[62] Xi R, Xie T 2005 Stem cell self-renewal controlled by chromatin remodeling factors. Science 310:1487–1489. doi:10.1126/science.1120140. 16322456

[63] Eissenberg JC, Wong M, Chrivia JC 2005 Human SRCAP and *Drosophila melanogaster* DOM are homologs that function in the notch signaling pathway. Mol Cell Biol 25:6559–6569. doi:10.1128/MCB.25.15.6559-6569.2005. 16024792 PMC1190335

[64] Young AP, Schlisio S, Minamishima YA, Zhang Q, Li L, Grisanzio C, Signoretti S, Kaelin WG Jr 2008 VHL loss actuates a HIF-independent senescence programme mediated by Rb and p400. Nat Cell Biol 10:361–369. doi:10.1038/ncb1699. 18297059

[65] Hsouna A, Nallamothu G, Kose N, Guinea M, Dammai V, Hsu T 2010 *Drosophila* von Hippel-Lindau tumor suppressor gene function in epithelial tubule morphogenesis. Mol Cell Biol 30:3779–3794. doi:10.1128/MCB.01578-09. 20516215 PMC2916397

[66] Dong S, Kantor AM, Lin J, Passarelli AL, Clem RJ, Franz AW 2016 Infection pattern and transmission potential of chikungunya virus in two New World laboratory-adapted *Aedes aegypti* strains. Sci Rep 6:24729. doi:10.1038/srep24729. 27102548 PMC4840389

[67] Nainu F, Tanaka Y, Shiratsuchi A, Nakanishi Y 2015 Protection of insects against viral infection by apoptosis-dependent phagocytosis. J Immunol 195:5696–5706. doi:10.4049/jimmunol.1500613. 26546607

[68] Lamiable O, Arnold J, de Faria I, Olmo RP, Bergami F, Meignin C, Hoffmann JA, Marques JT, Imler JL 2016 Analysis of the contribution of hemocytes and autophagy to *Drosophila* antiviral immunity. J Virol 90:5415–5426. doi:10.1128/JVI.00238-16. 27009948 PMC4934735

[69] Tassetto M, Kunitomi M, Andino R 2017 Circulating immune cells mediate a systemic RNAi-based adaptive antiviral response in *Drosophila*. Cell 169:314–325.e13. doi:10.1016/j.cell.2017.03.033. 28388413 PMC5730277

[70] Parikh GR, Oliver JD, Bartholomay LC 2009 A haemocyte tropism for an arbovirus. J Gen Virol 90:292–296. doi:10.1099/vir.0.005116-0. 19141437

[71] Hilton L, Moganeradj K, Zhang G, Chen YH, Randall RE, McCauley JW, Goodbourn S 2006 The NPro product of bovine viral diarrhea virus inhibits DNA binding by interferon regulatory factor 3 and targets it for proteasomal degradation. J Virol 80:11723–11732. doi:10.1128/JVI.01145-06. 16971436 PMC1642611

[72] Rodriguez-Andres J, Rani S, Varjak M, Chase-Topping ME, Beck MH, Ferguson MC, Schnettler E, Fragkoudis R, Barry G, Merits A, Fazakerley JK, Strand MR, Kohl A 2012 Phenoloxidase activity acts as a mosquito innate immune response against infection with Semliki Forest virus. PLoS Pathog 8:e1002977. doi:10.1371/journal.ppat.1002977. 23144608 PMC3493465

[73] Ulper L, Sarand I, Rausalu K, Merits A 2008 Construction, properties, and potential application of infectious plasmids containing Semliki Forest virus full-length cDNA with an inserted intron. J Virol Methods 148:265–270. doi:10.1016/j.jviromet.2007.10.007. 18054090 PMC7172237

[74] Ongus JR, Roode EC, Pleij CW, Vlak JM, van Oers MM 2006 The 5′ non-translated region of Varroa destructor virus 1 (genus *Iflavirus*): structure prediction and IRES activity in *Lymantria dispar* cells. J Gen Virol 87:3397–3407. doi:10.1099/vir.0.82122-0. 17030876

[75] Schnettler E, Sterken MG, Leung JY, Metz SW, Geertsema C, Goldbach RW, Vlak JM, Kohl A, Khromykh AA, Pijlman GP 2012 Noncoding flavivirus RNA displays RNA interference suppressor activity in insect and mammalian cells. J Virol 86:13486–13500. doi:10.1128/JVI.01104-12. 23035235 PMC3503047

[76] Anderson MA, Gross TL, Myles KM, Adelman ZN 2010 Validation of novel promoter sequences derived from two endogenous ubiquitin genes in transgenic *Aedes aegypti*. Insect Mol Biol 19:441–449. doi:10.1111/j.1365-2583.2010.01005.x. 20456509 PMC3605713

[77] Livak KJ, Schmittgen TD 2001 Analysis of relative gene expression data using real-time quantitative PCR and the 2^−ΔΔ^*CT* method. Methods 25:402–408. doi:10.1006/meth.2001.1262. 11846609

[78] Taylor SC, Nadeau K, Abbasi M, Lachance C, Nguyen M, Fenrich J 2019 The ultimate qPCR experiment: producing publication quality, reproducible data the first time. Trends Biotechnol 37:761–774. doi:10.1016/j.tibtech.2018.12.002. 30654913

