# Supplementary material for: The Aedes aegypti Domino Ortholog p400 Regulates Antiviral Exogenous Small Interfering RNA Pathway Activity and ago-2 Expression
Source: mSphere. 2020 Apr 8;5(2):e00081-20. doi: 10.1128/mSphere.00081-20 (PMC7142294; doi:10.1128/mSphere.00081-20)
Supplement: TABLE S1 [file mSphere.00081-20-st001.docx]

**Table S1**

| **Primer** | **Sequence** | **Use** |
| --- | --- | --- |
| dseGFP-T7 FOR | **GTAATACGACTCACTATAGGG**GGCGTGCAGTGCTTCAGCCGC | dsRNA |
| dseGFP-T7 REV | **GTAATACGACTCACTATAGGG**GTGGTTGTCGGGCAGCAGCAC | dsRNA |
| dslacZ-T7 FOR | **TAATACGACTCACTATAGGG**GTCGCCAGCGGCACCGCGCCTTTC | dsRNA (*in vivo*) |
| dslacZ-T7 REV | **TAATACGACTCACTATAGGG**CCGGTAGCCAGCGCGGATCATCGG | dsRNA (*in vivo*) |
| dsp400(AAEL001440)-T7 FOR | **GTAATACGACTCACTATAGGG**GTAGCAGCTACGGGAATGAC | dsRNA |
| dsp400(AAEL001440)-T7 REV | **GTAATACGACTCACTATAGGG**ACTCTGCCGACACTAGTTTTC | dsRNA |
| S7 QFOR (23) | CCAGGCTATCCTGGAGTTG | RT-qPCR |
| S7 QREV (23) | GACGTGCTTGCCGGAGAAC | RT-qPCR |
| p400KDeff qFOR | CCTGGGGAAAACTAGTGTCGG | RT-qPCR  KD efficiency |
| p400KDeff qREV | TGTTTGTCTTTGATTGGCCTGTT | RT-qPCR  KD efficiency |
| p400 QFOR | GGAACCAGTCCAGCCATGAA | RT-qPCR |
| p400 QREV | CGATCGCTCCTGCATTTGTG | RT-qPCR |
| Ago-2 QFOR (23) | GGCTGCTCACCCAATGTATCAAGA | RT-qPCR |
| Ago-2 QREV (23) | AACCGTTCGTTTTGGCGTTGAT | RT-qPCR |
| SFV-F (#) | CGCATCACCTTCTTTTGTG | RT-qPCR |
| SFV-R (#) | CCAGACCACCCGAGATTTT | RT-qPCR |
| Dcr-2 QFOR | ACCCACGTGTAATCGGTCTT | RT-qPCR |
| Dcr-2 QREV | CGGTAGCAATTGTCGCGTTA | RT-qPCR |

# Breakwell L, Dosenovic P, Karlsson Hedestam GB, D'Amato M, Liljestrom P, et al. 2007. Semliki Forest virus nonstructural protein 2 is involved in suppression of the type I interferon response. J Virol 81: 8677-8684.

For reference (23), please see article references.
