# Supplementary figures and images for: The Aedes aegypti Domino Ortholog p400 Regulates Antiviral Exogenous Small Interfering RNA Pathway Activity and ago-2 Expression
Source: mSphere. 2020 Apr 8;5(2):e00081-20. doi: 10.1128/mSphere.00081-20 (PMC7142294; doi:10.1128/mSphere.00081-20)

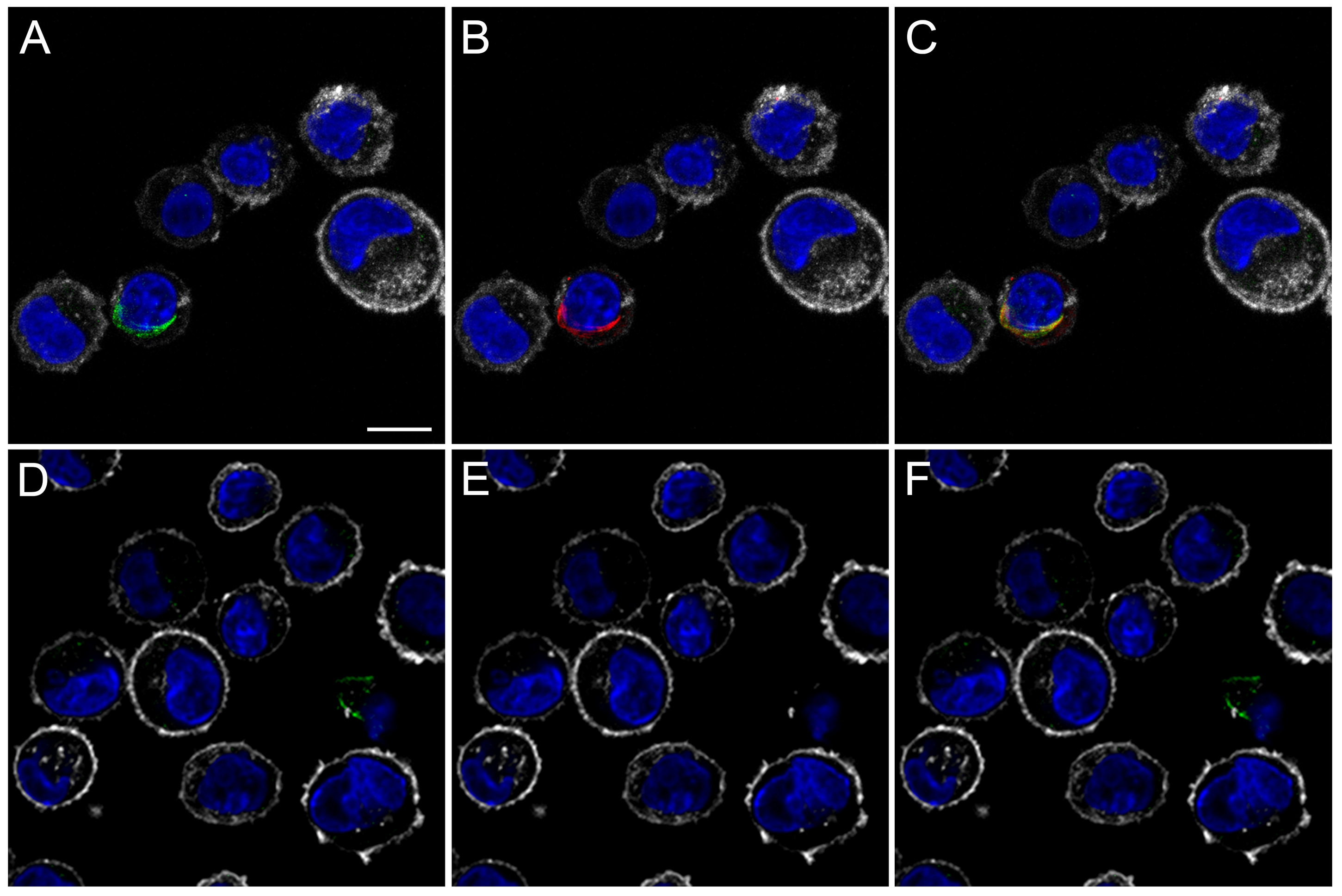

Supplement: FIG S3 [file mSphere.00081-20-sf003.tif]

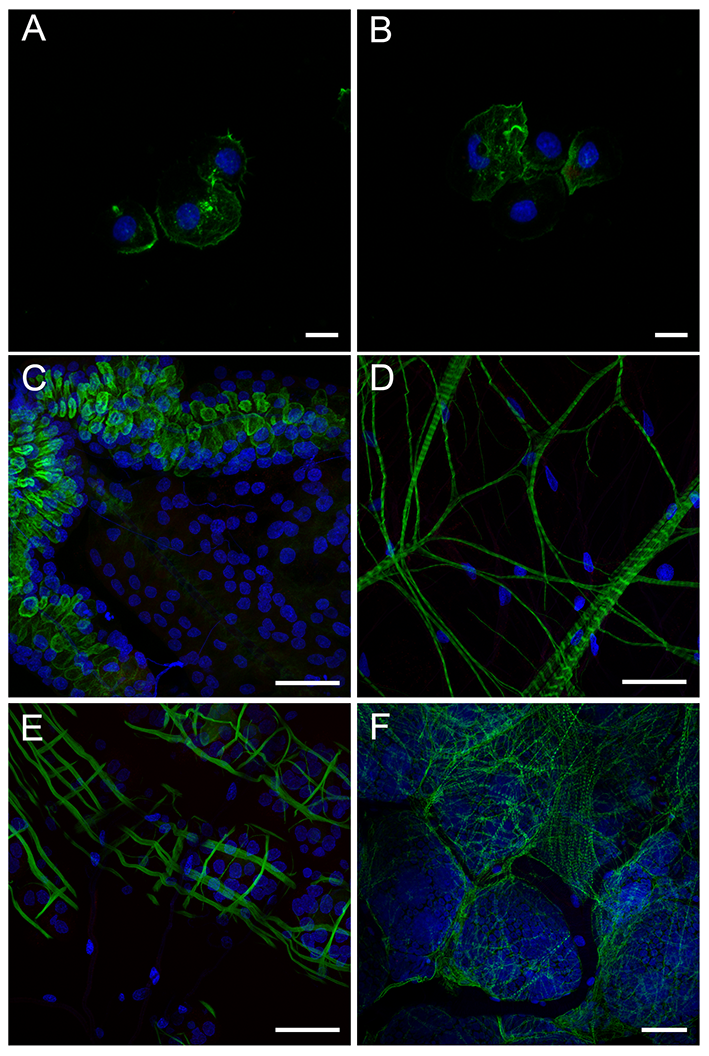

Supplement: FIG S4 [file mSphere.00081-20-sf004.tif]
